# Impacted Metallic Spring Requiring Cervical Esophagotomy: A Case Report and Review of the Literature on Foreign Body Removal

**DOI:** 10.1155/2017/5468131

**Published:** 2017-12-06

**Authors:** Patrick Stoner, Eric Hilgenfeldt, Alexander Schlachterman

**Affiliations:** ^1^Department of Internal Medicine, University of Florida Health, 1600 SW Archer Rd, Gainesville, FL 32610, USA; ^2^Department of Internal Medicine, Division of Gastroenterology, Carolinas Medical Center, 1000 Blythe Blvd, Charlotte, NC 28203, USA; ^3^Department of Internal Medicine, Division of Gastroenterology & Hepatology, Thomas Jefferson University, 833 Chestnut St., Philadelphia, PA 19107, USA

## Abstract

Foreign body ingestion is a commonly encountered problem and can cause significant morbidity and mortality. When removal of a foreign body from the upper gastrointestinal tract is indicated, endoscopy is the modality of choice and has a high reported success rate. However, in less than 1% of cases, endoscopic removal of a foreign body is unsuccessful and surgical intervention is necessary. We report a unique case of a large, sharp metallic spring swallowed by an incarcerated patient which subsequently became lodged in his upper thoracic esophagus. This spring was unable to be removed endoscopically due to risk of perforation and cervical esophagotomy was needed for its successful removal, illustrating the limitations of endoscopic techniques in removal of foreign bodies and the role surgical intervention has in these rare instances.

## 1. Introduction

Foreign body ingestion, defined as objects swallowed accidentally or intentionally or objects swallowed naturally when taking medication or food, is most commonly seen in the pediatric population in children between 6 months and 6 years [1, 2]. In adults, foreign body ingestion is mostly seen in specific high-risk groups of patients, including mentally impaired individuals, edentulous individuals, those with underlying esophageal pathology (i.e., esophageal ring, stricture, and malignancy), those under the influence of alcohol, patients with psychiatric disorders, and prisoners seeking secondary gain by access to a medical facility [1, 3, 4]. In the US alone, foreign body ingestion is a common problem and accounts for 1500 fatalities annually [1].

Management of foreign body ingestion is dependent upon several factors, including patient comorbidities, technical skills of the endoscopist, foreign body type, shape, size, impaction time, and anatomic site of entrapment in the gastrointestinal (GI) tract [1, 2, 5, 6]. In roughly 80% of cases of all foreign body ingestion, the object will pass spontaneously through the GI tract. However, in the remaining 20% of cases, retrieval of foreign bodies via esophagogastroduodenoscopy (EGD) is indicated in order to avoid serious complications, which can include perforation, necrosis, retropharyngeal abscess, mediastinitis, and fistulization [2, 3, 7]. Endoscopic removal of foreign bodies has a high success rate and low complication rates [5, 8]. However, when endoscopic removal is unsuccessful or contraindicated (as in our case), morbidity and mortality can be high [7]. In these rare instances (less than 1% of cases), surgery is indicated. We present a unique case of foreign body ingestion in which endoscopic retrieval was attempted but aborted in favor of cervical esophagotomy in order to prevent perforation or mediastinitis and provide a brief review of the literature regarding foreign body removal from the upper GI tract.

## 2. Case Presentation

**Figure 1:**
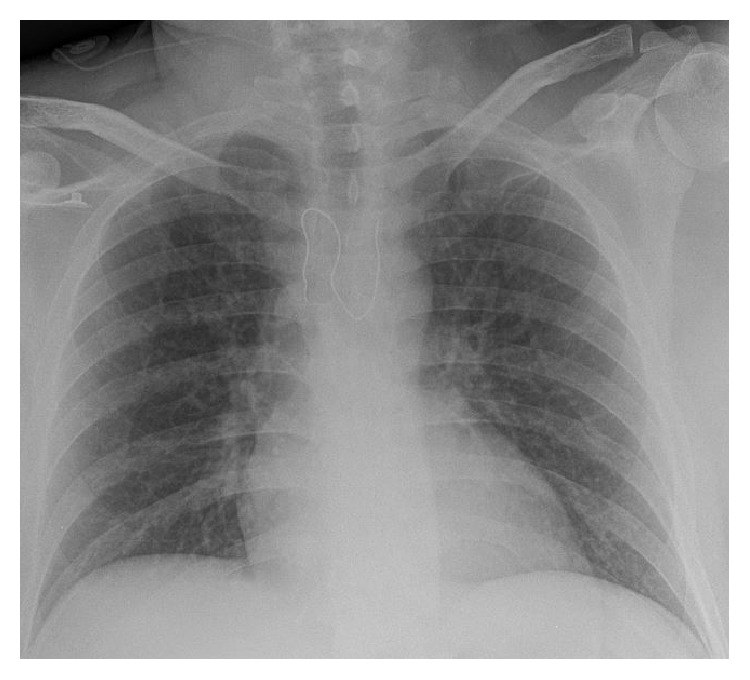
Chest X-ray showing a curvilinear metallic object overlying the upper mediastinum.

**Figure 2:**
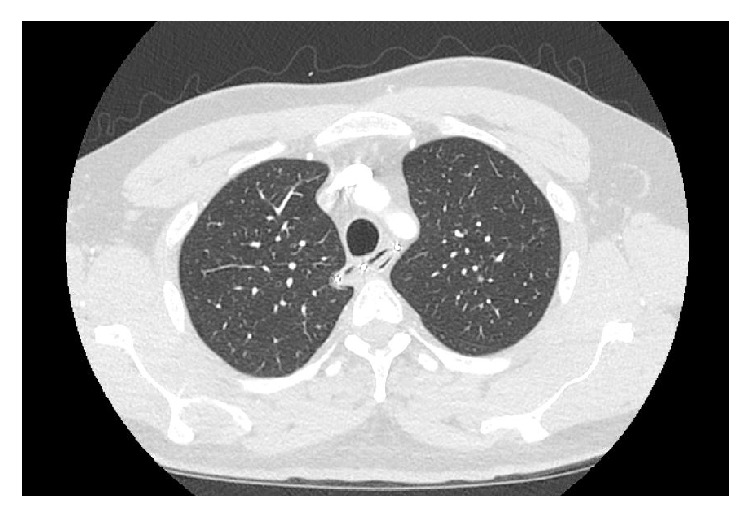
CT of the chest abdomen and pelvis showing metallic foreign body lodged in the upper esophagus without evidence of perforation.

An incarcerated 43-year-old male with noncontributing past medical history presented to the Emergency Department (ED) via Emergency Medical Services (EMS) complaining of dysphagia and pain in his urethra. EMS explained that, at about 8:00 PM that night, the patient wrapped a piece of a metal spring from his jail bed in toilet paper and then swallowed it. He also inserted components of a ballpoint pen into his urethra. On presentation in the ED, his vital signs were stable and labs were within normal limits. A chest X-ray (Figure 1) showed a curvilinear metallic object overlying the upper mediastinum. The patient subsequently had a CT of the chest abdomen and pelvis which showed that the metallic foreign body was indeed lodged in the upper esophagus without evidence of perforation (Figure 2).

**Figure 3:**
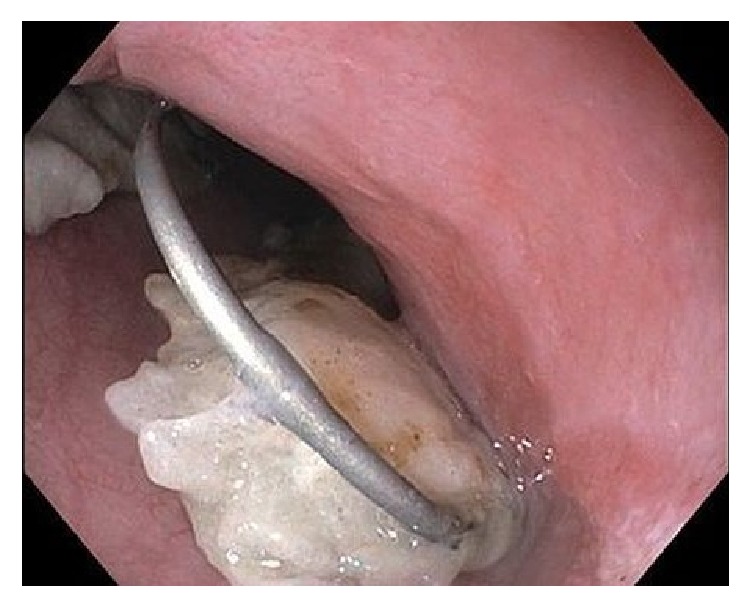
Metallic spring deployed in the upper thoracic esophagus 21 cm from the incisors.

**Figure 4:**
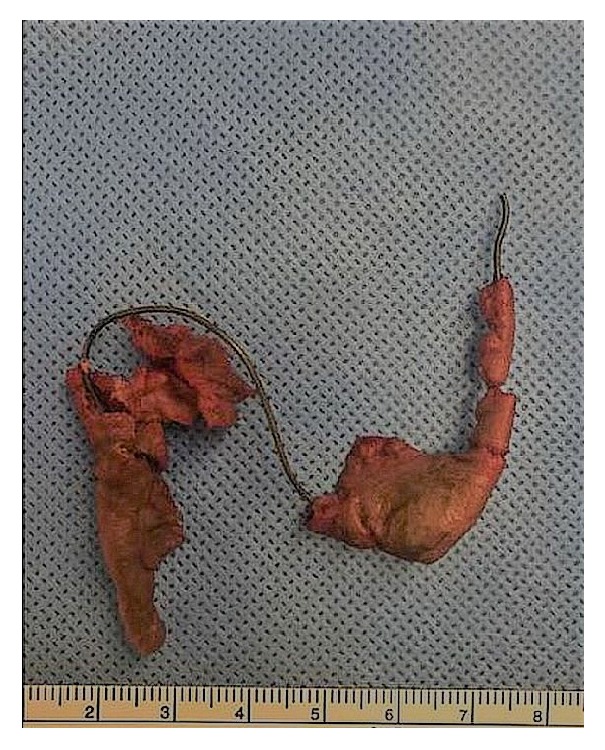
Metallic spring excised by cervical esophagotomy.

Esophagogastroduodenoscopy (EGD) was performed and revealed that the toilet paper wrapping that allowed for swallowing of the metallic spring had dissolved, causing the spring to deploy in the upper thoracic esophagus (like a spring-loaded trap) 21 cm from the incisors (Figure 3). Removal of the spring was initially attempted using rat tooth forceps and a protector hood. However, it was soon discovered that the sharp edges of the spring had impacted into the walls of the esophagus and therefore endoscopic removal of the spring was aborted due to risk of esophageal perforation. Surgery was consulted and the metallic spring (Figure 4) was removed from the upper thoracic esophagus via cervical esophagotomy.

A pelvic X-ray done in the ED was negative and the patient later underwent cystoscopy under general anesthesia with successful removal of the pen from the penoscrotal junction by urology. The patient tolerated all procedures well without complications and was transported back to prison after a two-day hospital stay.

## 3. Discussion

Most ingested foreign bodies pass spontaneously through the GI tract within 4–6 days and therefore can be managed by means of close observation of the patient's stools [1, 2]. This approach is indicated for blunt, short (<6 cm), and narrow (<2.5 cm in diameter) foreign bodies, especially once they have passed the pylorus [1, 2]. Urgent EGD is indicated for nonoccluding esophageal foreign bodies and ingestion of magnets and for objects that are >6 cm in length [2]. Emergent EGD is recommended in cases of complete esophageal occlusion causing salivary pooling due to the risk of aspiration and/or pressure necrosis when the object ingested has sharp points or edges (to avoid risk of perforation, as in our case) and when a battery has been ingested (due to risk of necrosis and fistula formation) [1, 2, 4, 6, 8].

Reported rates of successful endoscopic removal of foreign bodies are as high as 98%, with minimal to no complications [1, 8]. The preferred endoscopic method is using a flexible forward-viewing endoscope under conscious sedation or general anesthesia [3, 8]. There are several types of endoscopic tools used to extract foreign bodies and the tool of choice depends on the characteristics of the foreign body. More commonly used tools include rat tooth forceps, alligator forceps, retrieval nets, protector hoods, and overtubes [6, 7]. These last two tools are indicated in the removal of sharp objects. In our case, rat tooth forceps and a protector hood were used in attempts to remove the metal spring. Use of an overtube was considered but was not utilized since it was determined at that point that both ends of the spring were lodged in the esophageal wall. A therapeutic dual scope for potential grasping of both ends of the deployed spring could also not be considered for this reason. Thus, surgical removal was indicated.

When a foreign body is impacted in the esophagus, it is usually at an area of anatomic or physiologic narrowing, such as the upper esophageal sphincter, lower esophageal diaphragmatic sphincter, or mid-esophageal site of extrinsic compression by the aorta and left main bronchus (as in our case) [5, 7]. In addition to anatomy, other factors exist which can portend the probability of successful foreign body endoscopic removal, including shape and size of objects, impaction time, underlying diseases, and skill of the endoscopists [2, 5]. A retrospective review of 885 patients treated for suspected foreign body ingestion found that older age (>70 years), location of foreign body in the upper esophagus, larger size (maximal diameter > 30 mm), and longer impaction time (>40 hours) were significant risk factors predicting conversion to surgery after inability to remove the foreign body endoscopically [5].

In addition to having two out of the four above-mentioned risk factors (impaction in upper esophagus and object size > 30 mm) for endoscopic failure and the subsequent rare need for conversion to surgery, our case is unique given the characteristics of the object ingested and the events that ensued after ingestion. The foreign body, a piece of metallic spring apparently broken off from the patient's jail bed, was large (~7 cm) and sharp, requiring the patient to wrap it in toilet paper in order to be able to swallow it. When the toilet paper dissolved, the spring deployed and both ends wedged into the esophageal wall on opposite sides. This was seen on endoscopy and it was determined that manipulating the spring (i.e., grasping it with rat tooth forceps and rotating it or pulling it cephalad) would likely cause perforation. We were able to find only two case reports [9, 10] describing ingested metallic spring-like objects, both of which were in pediatric population. One of these did require esophagotomy after failed endoscopic removal [10].

Overall, cases of foreign body ingestion requiring cervical or thoracic esophagotomy are rare. A systematic review by Heger et al. [7] identified 11 publications describing the case reports of a total of 29 patients who underwent esophagotomy for foreign body removal. In all cases, surgery was deemed necessary due to risk of complications (i.e., perforation) and/or after failed attempts to extract the foreign body were made endoscopically. There were no deaths reported and the overall complication rate in these cases was 17.2%, which the authors deemed justifiable given the high risks of no surgical intervention [7].

In summary, when removal of a foreign body from the upper GI tract is indicated, endoscopy remains the initial modality of choice and is safe and effective [1, 4, 5, 8]. However, in rare cases of unsuccessful endoscopic foreign body removal or when risk of complications such as esophageal perforation is deemed high, surgical resection is indicated. We report an interesting case of foreign body ingestion in which endoscopic retrieval was aborted in favor of surgical resection. This case highlights the limitations of endoscopic removal of certain foreign bodies and illustrates the safe and effective surgical alternative of esophagotomy.
